# Prevalence of group B streptococcus colonization in Iranian pregnant women: A systematic review and meta-analysis

**DOI:** 10.18502/ijrm.v16i12.3679

**Published:** 2019-01-28

**Authors:** Mohammad Hossein YektaKooshali, Masoud Hamidi, Seyed Mohammad Taghi Razavi Tousi, Iraj Nikokar

**Affiliations:** ^1^Student Research Committee, School of Nursing-Midwifery and Paramedicine, Guilan University of Medical Sciences, Rasht, Iran.; ^2^Medical Biotechnology Research Center, Guilan University of Medical Sciences, Rasht, Iran.

**Keywords:** * Intracytoplasmic sperm injection*, * Empty follicle syndrome*, * HCG.*

## Abstract

**Background:**

Group B Streptococcus (GBS) is an important pathogen in newborns and pregnant women.

**Objective:**

The present study was carried out to estimate the prevalence of GBS colonization in pregnant women in Iran.

**Materials and Methods:**

This systematic review and meta-analysis was based on Preferred Reporting Items for Systematic Reviews and Meta-Analysis guideline using the national databases including Society for Information Display, Magiran, Irandoc, Iran Medex, and international databases including MEDLINE, Web of Science, Scopus, PubMed, Science-Direct, Cochrane, Embase, Elton Bryson Stephens Company, Centre for Evidence-Based Medicine, Cumulative Index to Nursing and Allied Health Literature, and Google Scholar, published by 01/30/2017. The I${}^{2}$ index was used to measure heterogeneity between the studies.

**Results:**

In a total of 667 documents, 30 (4.49%) were selected. In this study, the prevalence of GBS colonization in 10090 Iranian pregnant women was calculated as 13.65% [confidence interval (CI): 95%: 10.56–17.45]. Based on geographic region, 24.63% [CI: 95%: 11.52–45.06] in the West and 8.75% [CI: 95%: 6.43–11.8] in the East were the highest and lowest areas in Iran, respectively, and were statistically significant (*p* = 0.001). Also, with regards to swapping sampling area, Vaginal with 11.96%, Vaginal and Rectal with 13.62%, and Anal and Vaginal with 25.63% were the least to the greatest, respectively, and were statistically significant (*p* = 0.001).

**Conclusion:**

Therefore, based on the recommendation of Centers for Disease Control and Prevention as reported by the Ministry of Health and Medical education, early diagnosis, and screening of high-risk women should be done at 35–37 weeks of pregnancy.

## 1. Introduction

Group B Streptococcus (GBS) as an important pathogen in newborns and pregnant women emerged as the leading cause of neonatal infections since 1970 (1–3). *Streptococcus agalactiae* is a gram-positive anaerobic, spherical, encapsulated, solid growth, catalase-negative, and non-motile bacterium. Its anti-phagocytic polysaccharide capsule is the most important factor of pathogenicity. This bacterium is an intestinal normal flora in some humans and may colonize secondary sites such as the vagina in some women (4–6). GBS colonization occurs in the genital tract of 10–30% of pregnant women and is without symptoms. *Streptococcus agalactiae* can cause infection incidents in the urethra, chorioamnionitis, endometritis, septicemia, infectious abortion, osteomyelitis, pyelonephritis, puerperal fever, rupture of membranes, and cystitis in women. During birth, especially as the baby passes through the birth canal, it can cause diseases including sepsis, pneumonia, septic arthritis, delayed infections, urinary and gastrointestinal tract disorders, and cellulite in infants, which leads to deaths in 10–20% of cases. Even if the infants survive, they may be mentally retarded and experience vision problems (5–12).

Bacterium acquisition by the infant leads to the colonization of the skin or mucous membranes, which is transferred from infected mothers to 15–50% of infants and only progresses in 1–3% of them, leading to the aforementioned diseases (11). The disease early-onset in days before the end of 1 week (0–7 days) emerges as septicemia willing to pneumonia, and in the case of late-onset (after 1 week (days 7–90)) in infants manifests as septicemia willing to meningitis, which despite antibiotic therapy, has accounted for a high mortality (13, 14). Colonization of this bacterium can be transient, intermittent, or chronic, and its incidence varies in different geographical areas (15). Studies have reported GBS colonization to be less in women of over 20 years old and with multiple pregnancies, and more in women with multiple sexual partners, suffering from diabetes mellitus, suffering from cancer, and patients with weakened immune systems (5–8).

Also, social conditions, microbiology detection methods, different sampling location, and age of the study population could also cause variations in the incidence of GBS colonization in pregnant women (16). According to the guidelines of the Centers for Disease Control and Prevention (CDC) based on screening of pregnant women and risk factors, the prevalence of *S. agalactiae* infections has decreased in infants. To prevent the occurrence, this center has embarked on the screening of all pregnant women with a history of urinary tract infection, prom, diabetes mellitus, puerperal fever, and premature delivery in weeks 35–37 as its agenda (17–23).

One of the main objectives of meta-analysis studies is to combine existing studies to increase the sample size because of the increased number of relevant studies. Thus, this can reduce the difference in existing parameters and also the confidence interval; finally, the result can lead to solving the aforementioned problems with the last procedure (24, 25). Absolutely, these studies are a vitally important link between research studies and decision-making at the bedside of the patient (26–28). Studies have reported GBS infection to range from 3 to 75% in Iran (5, 6, 8, 21, 23, 28–36). Moreover, in Iran, CDC's prevention orders related to these bacteria are not implemented as a codified program.

As a result of the aforementioned cases, the severity and spread of GBS infection, the controversy in the reported incidence of GBS, lack of access to the global community of these factors by pregnant women in Iran, as well as the expression of the final conclusions for policymaking and proper management planning in the country, a systematic review of all published documents through valid databases and combination of data using meta-analysis method were implemented for overall estimation of the prevalence rate of GBS in pregnant women in Iran.

## 2. Materials and Methods

The present study is the first systematic and meta-analysis review with the aim of investigating the prevalence of colonization of GBS in Iranian pregnant women until January 30, 2017. The study was conducted in accordance with the PRISMA (Preferred Reporting Items for Systematic Reviews and Meta-analyses) guidelines (37).

### Study selection

#### Inclusion and exclusion criteria

The main inclusion criterion of the study includes colonization rate of GBS in pregnant women in Iran. The exclusion criteria were: 1. lack of reference to the prevalence of GBS colonization in pregnant women; 2. little information; 3. lack of connection with the subject of the research; 4. non-random sample size; 5. qualitative articles and letters to the editor; 6. duplicate articles; 7. non-Iranian study community.

#### Search strategy and study selection

**Figure 1:**
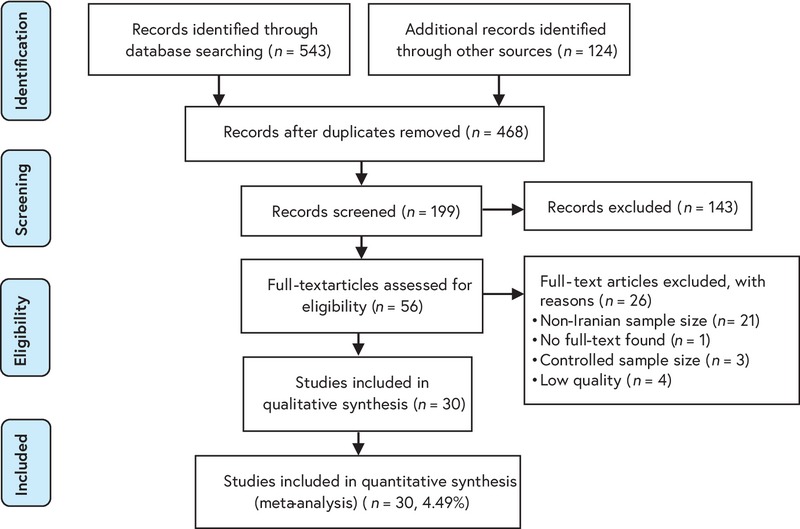
A flow diagram following the PRISMA.

The results of this study are based on articles published in national and international journals, dissertations, and reference sites. A review of related English and Persian literature was performed in the national databases Society for Information Display, Magiran, Irandoc, IranMedex, and international databases MEDLINE, Web of Sciences, Scopus, PubMed, Science-Direct, Cochrane, Embase, Elton Bryson Stephens Company, Centre for Evidence-Based Medicine, Cumulative Index to Nursing and Allied Health Literature, and Google Scholar. Search for articles was conducted using the Persian keywords and English equivalent in accordance with its Mesh after full consideration to some published primary studies: `group B Streptococcus', `*Streptococcus agalactiae*', `colonization', `Prevalence', `Incidence', `Epidemiology', `symptom', `neonatal infection', `pregnant women', `Iran', and all possible combinations of words using Boolean operators in combination for the English language bases. Also, the manual search, using a checklist of articles was identified and was carried out to find additional articles (Figure 1). The searched syntax of the PubMed database was added as an example in the appendix.

The important point in searching databases was to perform searches with high sensitivity (High Sensitive Searching) and also the search was performed by a senior researcher and expert in the field of searching databases. The entire process of research including, search, selection of studies, quality assessment of studies, and data extraction was performed independently by two researchers to avoid publication bias, and third researcher will be making the final assessment in the event of conflictions. After the search, EndNote${}^{TM}$ software, were used to find duplicates. Also manual searching was done by reviewing the reference list of relevant articles.

### Quality assessment

**Figure 2:**
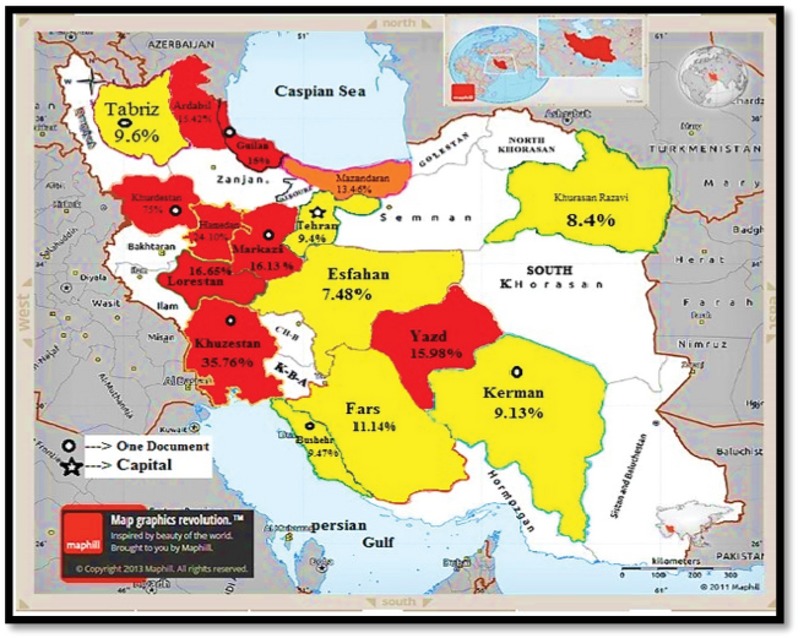
Map of Iran showing selected studies based on random effects meta-analyses. Red Color: Higher prevalence of GBS colonization in Iranian pregnant women in comparison to total prevalence. Orange Color: Same prevalence of GBS colonization in Iranian pregnant women in comparison to total prevalence. Yellow Color: Lower prevalence of GBS colonization in Iranian Pregnant women in comparison to total prevalence. Source: Map of Iran powered by http://www.maphill.com/, which is marked as `free' and licensed under a Creative Commons Attribution Non-Derivative License (CC BY-ND)("© Maphill / CC BY-ND").

After verifying the relevant studies, STROBE (Strengthening the Reporting of Observational Studies in Epidemiology) checklist were used to evaluating selected articles by researchers (38). This checklist contains 22 items and evaluates various aspects of the methodology. The researchers divided the articles, in terms of quality, into three categories: Low Quality (0–15), medium (16–30), and high (31–44); the articles with at least 16 points entered the quantitative meta-analysis stage (Table I, Figure 2).

### Data extraction

First, a checklist was designed on the basis of the objectives and by studying other resources available. The designed checklist included items, such as Authors, Year, Place, Sample Size, Gestation (wk), Regions of Where Mucus Swap Taken, Positive GBS Cultures, Prevalence (%), which were extracted by two researchers independently and blind to the author's name, institution, and journal. When required, additional information and raw data were asked by contacting the corresponding author (Table I).

### Statistical analysis

In each study, after taking into account the prevalence of GBS colonization in pregnant women in Iran as a binomial probability distribution, its variance was calculated by the binomial distribution, and the heterogeneity of the studies was evaluated using the Q sample test and index I${}^{2}$. Due to the heterogeneity of the studies, the random effects model was used to combine the results of the studies. Data analysis was conducted using meta-analysis specialized software – Comprehensive Meta-Analysis Ver.2, and a significance level of tests was considered *p* < 0.05.

## 3. Results

**Figure 3:**
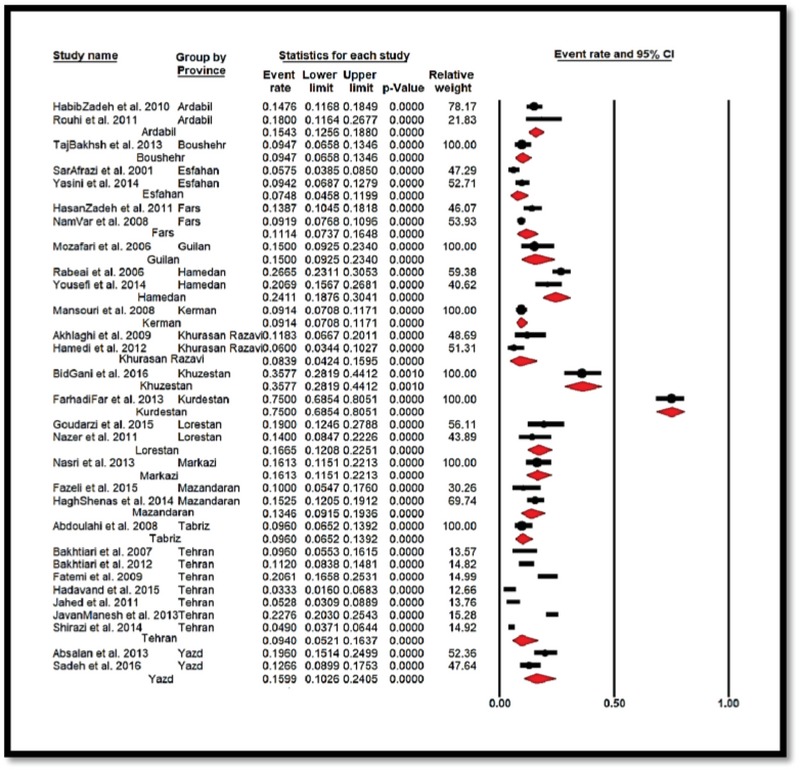
Forest Plot showing the prevalence of GBS colonization in Iranian pregnant women sub-grouped according to the province of papers that were entered to meta-analysis.

**Figure 4:**
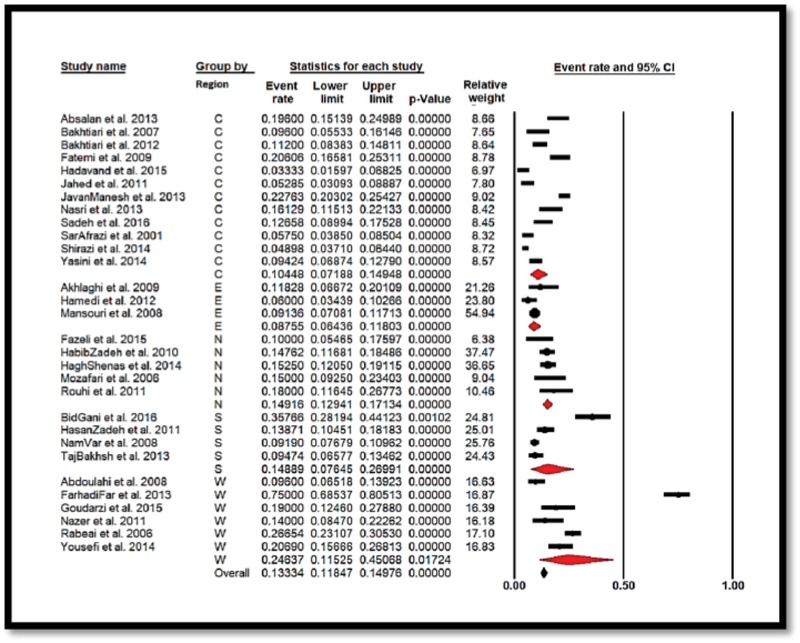
Forest plot showing the prevalence of *Streptococcus agalactiae* colonization in Iranian pregnant women sub-grouped by region (C = Center, E = East, N = North, S = South, W = West).

**Figure 5:**
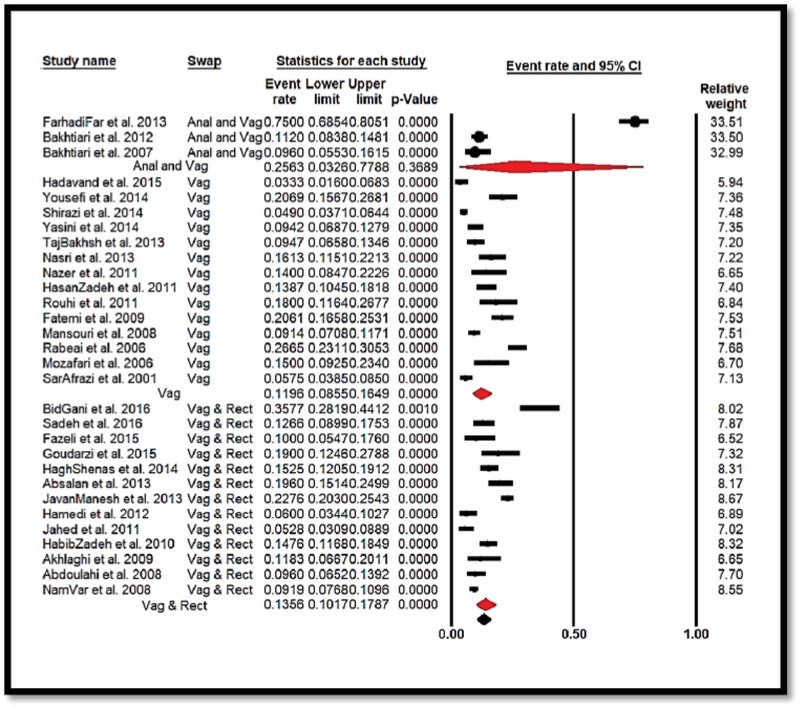
Forest plot 3 showing the prevalence of *Streptococcus agalactiae* colonization in Iranian pregnant women sub-grouped by regions where mucus swap took (Anal & Vaginal, Vaginal, and Vaginal & Rectal).

In this systematic study, based on the searches conducted, 667 papers were identified, and after a final review and assessment in accordance with the checklist, 30 cases (4.49%) in a total of 10090 Iranian pregnant women entered the list (Table I). The results of the systematic review and meta-analysis were calculated as shown in Figures 3–5.

GBS colonization prevalence in Iranian pregnant women was calculated as 13.65% [confidence interval (CI): 95%: 10.56–17.45]. The highest and lowest prevalence was related to studies conducted in Kurdistan and Esfahan with 75% [CI: 95%: 68.53–80.51], and 7.48% [CI: 95%: 4.57–11.99], respectively (Figure 3).

From the results of the meta-analysis, the prevalence of GBS colonization in Iranian pregnant women based on the geographic region was 24.63% [CI: 95%: 11.52–45.06] in the West and 8.75% [CI: 95%: 6.43–11.8] in the East. Thus, these values were, respectively, the highest and lowest areas in Iran, and were statistically significant (*p* = 0.001) (Figure 4). Also, based on the swap sampling area in the study and the prevalence of GBS colonization in Iranian pregnant women, respectively, Vaginal with 11.96% [CI: 95%: 8.55–16.49], Vaginal and Rectal with 13.62% [CI: 95%: 10.16–17.86], and Anal and Vaginal with 25.63% [CI95%: 3.26–77.87] were from the least to the greatest, and were found to be statistically significant (*p* = 0.001) (Figure 5).

**Figure 6:**
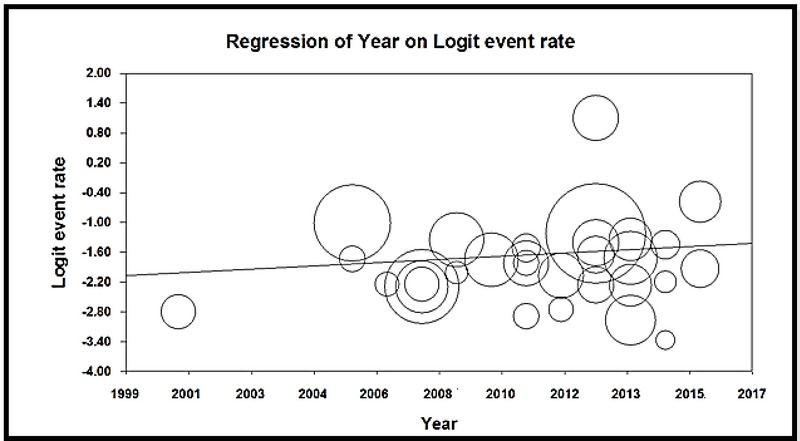
The meta-regression diagram of the prevalence of *Streptococcus agalactiae* colonization in Iranian pregnant women based on the year of study. The greater circle shows the greater sample size number.

**Figure 7:**
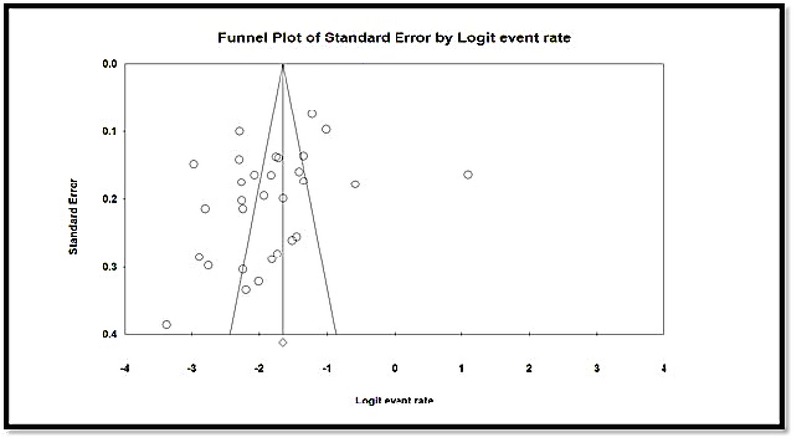
The Funnel Plot of the prevalence of *Streptococcus agalactiae* colonization in Iranian pregnant women.

According to the meta-regression graph, GBS colonization prevalence in pregnant women in Iran increased with increase in study years, and a statistically significant difference was observed (*p* = 0.001) (Figure 6). Based on the result of Begg's and Egger's tests, the probability of the publication bias was not statistically significant (P = 0.09353 and P = 0.10620, respectively) (Figure 7).

**Table 1:** Characteristics of articles included in the meta-analysis.


Author (Ref. no.)	**Place**	**Year**	**Multi/Single Center of where data were taken**	**Gestation (Wks.)**	**Regions of Where Mucus Swap Taken**	**Sample Size**	**Positive GBS Cultures**	**Prevalence (%)**
Absalan et al. (39)	Yazd	2013	Multicenter	NA	Vag* & Rect*	250	49	19.6
Bakhtiari et al. (29)	Tehran	2007	Single Center	35–37	Anal, Vag	125	12	9.6
Jahed et al. (22)	Tehran	2011	Single Center	35–37	Vag, Rect	246	13	5.28
Javan Manesh et al. (17)	Tehran	2013	Multicenter	35–37	Vag, Rect	1028	234	22.76
Sadeh et al. (40)	Yazd	2016	Single Center	NA	Vag, Rect	237	30	12.65
Fatemi et al. (12)	Tehran	2009	Single Center	NA	Vag	330	68	20.60
Hadavand et al. (41)	Tehran	2015	Single Center	35–37	Vag	210	7	3.33
Nasri et al. (4)	Arak	2013	Single Center	35–37	Vag	186	30	16.12
SarAfrazi et al. (42)	Kashan	2001	Single Center	≥ 35	Vag	400	23	5.75
Shirazi et al. (34)	Tehran	2014	Single Center	35–37	Vag	980	48	4.89
Yasini et al. (35)	Kashan	2014	Single Center	28–37	Vag	382	36	9.42
Bakhtiari et al. (23)	Tehran	2012	Single Center	28–38	Anal, Vag	375	42	11.2
Akhlaghi et al. (32)	Mashhad	2009	Single Center	34–37	Vag, Rect	93	11	11.82
Hamedi et al. (43)	Mashhad	2012	Single Center	38–40	Vag, Rect	200	12	6
Mansouri et al. (30)	Kerman	2008	Multicenter	35–37	Vag	602	55	9.13
Fazeli et al. (36)	Amol	2015	Multicenter	35–37	Vag, Rect	100	10	10
Habib Zadeh et al. (44)	Ardabil	2010	Multicenter	35–37	Vag, Rect	420	62	14.7619
Hagh Shenas et al. (5)	Babol	2014	Single Center	35–37	Vag, Rect	400	61	15.25
Amirmozafari et al. (14)	Rasht	2006	Single Center	28–37	Vag	100	15	15
Rohi et al. (33)	Ardabil	2011	Single Center	8–40	Vag	100	18	18
Bid Gani et al. (20)	Ahvaz	2016	Single Center	35–37	Vag, Rect	137	49	35.76
Jahromi et al. (45)	Shiraz	2008	Single Center	≥ 24	Vag, Rect	1197	110	9.18
Hassan Zadeh et al. (21)	Shiraz	2011	Multicenter	35–41.6	Vag	310	43	13.87
Taj Bakhsh et al. (18)	Boushehr	2013	Multicenter	≥ 35	Vag	285	27	9.47
Abdoulahi Fard et al. (46)	Tabriz	2008	Single Center	NA	Vag, Rect	250	24	9.6
Goudarzi et al. (47)	Khorramabad	2015	Multicenter	35–37	Vag, Rect	100	19	19
Kalantar et al. (48)	Sanandaj	2013	Multicenter	28–38	Anal, Vag	200	150	75
Nazer et al. (8)	Khorramabad	2011	Single Center	28–37	Vag	100	14	14
Rabiei et al. (19)	Hamedan	2006	Multicenter	> 20	Vag	544	145	26.65
Yousefi Mashouf et al. (28)	Hamedan	2014	Multicenter	35–37	Vag	203	42	20.68

Note: *NA: Not available; Rect: Rectal; Vag: Vaginal; GBS: Group B streptococcus.

## 4. Discussion

Based on the systematic review and meta-analysis of the results of the studies, the prevalence of GBS colonization in Iranian pregnant women was estimated at 13.65% [CI: 95%: 10.56–17.45]. Thus, this prevalence in other developing countries, such as India, Turkey, Thailand, Kuwait, Saudi Arabia, and Nigeria is 2.3, 9.2, 12.9, 16.4, 13.9 and 19.5%, respectively (49). The difference in standards and laboratory diagnostic methods, health and medical policy of countries, preventive measures along ethnic differences, geographic region, differences in sampling locations, bacteriological methods for the detection of organisms, statistical differences in the study population, laboratory staff skill, and sex partners can be the reasons for the differences in other countries. Also, in a study by Turrentine and colleagues (40), the prevalence of GBS colonization and its recurrence during pregnancy in other five cohort studies that were meta-analyzed was 44.5% (512/1150) and a significant relationship was reported. Lower than the results of the present study, the prevalence of GBS Colonization has been reported to be more in developed countries, such as Italy (16.5%), Brazil (14.6%), Canada (11.6%), the UK (10.5%), Greece (6.6%), and Israel (6.5%) (12, 19–21, 39, 49–53).

Based on the results of the meta-analysis, the swap sampling area of the studies and GBS colonization prevalence in Iranian pregnant women were statistically significant (*p* = 0.001). The prevalence rate from low to high was, respectively, in the isolated Vaginal swap with 11.96%, Vaginal and Rectal with 13.62%, and Anal and Vaginal with 25.63%. The colonization of GBS in the vagina is about 2 times of the rectum (26, 27, 36). But since studies have examined Vaginal only or Vaginal and Rectal together, the information on each one cannot be separate from the other in the investigation of the prevalence of these issues in Iranian society. But the overall review of the prevalence in individual studies indicates a relatively increased frequency of GBS colonization of the swap isolated from anal relative to rectal and vaginal, and rectal to vaginal. In a study by Akhlaghi and co-workers (32), the prevalence of GBS colonization of the swap isolated from pregnant women with diabetes was reported to be more as compared with non-diabetic (almost double). This can be due to the fact that pregnant women with diabetes are at greater risk of infection (54).

In another review of the results of the meta-analysis, the prevalence of GBS colonization in pregnant women in Iran based on geographic region from low to high were, respectively, 8.75% in the East, 10.44% in the Central, South 14.89%, North 14.91%, and West 24.63%, and these were statistically significant (*p* = 0.001). In Iran, the different antibiotic resistance pattern and differences in the anti-geneity, imno-geneity, and pathogenicity of different serotypes in various geographical areas can be ascribed to several factors (6, 40, 55). Also, in an assessment conducted by Fazeli and colleagues (36), the prevalence of GBS colonization was reported to be more in rural pregnant women, and a significant relationship was reported. More contact with animals, livestock, and its products can be of effective factors in this regard (34).

According to the meta-regression graph, GBS colonization prevalence in pregnant women in Iran increased with an increase in the year of the study and was statistically significant (*p* = 0.001). Various factors, including the lack of care of pregnant women, and enforcement of prevention and screening programs can be important in the prevalence of this bacterium.

In a study conducted in the United States of America (USA), all isolates were sensitive to penicillin, ampicillin, and cefazolin, and resistances of 25.6% and 12.7% were reported to erythromycin and clindamycin, respectively (56). In another study, the antibiotic susceptibility of the bacteria was reported with disk diffusion method, as 100% of cases were resistant to penicillin and ampicillin, and 26.9% and 42.1% to erythromycin and clindamycin, respectively (26). In studies in Iran, Iranian sensitivity of GBS in pregnant women was obtained as 97.2% to ampicillin, 80.5% to erythromycin, 83.4% to clindamycin (35), and Cefalozin 76.5% (37). Therefore, according to studies on resistance and susceptibility behavior of antibiotics to GBS, penicillin has been selected for the prevention and treatment of GBS infections, which is usually given in early labor. Erythromycin and clindamycin are prescribed for pregnant women at risk of anaphylaxis instead of penicillin or cefazolin (57). While penicillin is a good choice for women who are not at the risk of anaphylaxis (10, 56, 58), nitrofurantoin can also be effective in the symptomatic and asymptomatic treatment of the bacteria *S. agalactiae*. In the case of drug resistance to these antibiotics, vancomycin can be used (36, 59). Fulfillment of orders recommended by CDC and the use of antibiotic prophylaxis in leading countries witnessed a 70% reduction in the prevalence of infection in infants (20, 60). This has also been confirmed in Iranian Studies (19, 28, 39, 40).

Given the high prevalence of colonization of GBS in pregnant women in Iran, screening all High-risk pregnant women in weeks 35–37, the antibiotic susceptibility situation, investigating the common serotypes, and steps required to develop a vaccine for the common serotypes, according to the statement of the CDC, are necessary measures for prevention that should be given priority by the Ministry of Health and Medical education. Also, CDC has introduced the culture of samples in selected broth and then culturing on selective agar, and finally using polymerase chain reaction (PCR) technique as the best method to select in vitro diagnostic tests (36, 61).

### Limitations

The heterogeneity rate (I2) was calculated as 95.71% in this research, which is in high level (I2 index less than 25% low, 25 to 75% average, and more than 75% higher heterogeneity (62)). Differences is expected because of different sampling and measured parameters in different populations.

• The insensitivity of national databases to operators `AND' and `OR' to search for the combination and the absence of services like Mesh subject heading to contain all related and same definition of words which were used in published papers.
• Failure to assess the prevalence of GBS colonization in Iranian pregnant women in separate locations of swaps supply due to the limited number of studies.
• Since the main medical centers are located in many cities and provinces of Tehran, not all women in the studies are from Tehran.
• No separation of rural and urban prevalence and frequency of GBS colonization in women.

## 5. Conclusion

According to the studied documents, the prevalence of GBS colonization in Iranian pregnant women in the west of Iran is much higher than other regions, and also no research has been done in some provinces in the west. It is therefore suggested that coherent and fresh studies be conducted in these areas. While Iranian women constitute half of the population of the country, today, disregarding this population to improve physical and mental health has become a problem from the viewpoint of researchers and activists of women's health of the country. Since women and mothers play an important role in family and community health, better management decisions for the prevention, screening, and treatment are recommended.

##  Conflict of Interest

The authors declare no conflicts of interest.

## Appendix

"*Streptococcus agalactiae*" [All Fields] OR "group B streptococcus" [All Fields] AND ("Iran"[MeSH Terms] OR "Iran"[All Fields]) AND (("epidemiology" [Subheading] OR "epidemiology" [All Fields] OR "prevalence" [All Fields] OR "prevalence" [MeSH Terms]) OR ("epidemiology" [Subheading] OR "epidemiology"[All Fields] OR "epidemiology" [MeSH Terms])) AND "Pregnant Women" [All Fields]
